# Sociodemographic, labor conditions, habits, lifestyles and diabetes *mellitus* in workers with subsistence jobs, Medellín-Colombia

**DOI:** 10.47626/1679-4435-2020-560

**Published:** 2021-02-11

**Authors:** María Osley Garzón-Duque, Fabio León Rodríguez-Ospina, Doris Cardona, Ángela María Segura-Cardona, María Camila Borbón, Ana María Zuluaga-Giraldo, José Ignacio Echeverri-Loor

**Affiliations:** 1 Universidad CES, Facultad de Medicina - Medellín (ANT), Colombia; 2 Universidad de Antioquia, Facultad Nacional de Salud Pública - Medellín (ANT), Colombia; 3 Universidad CES, Facultad de Medicina - Escuela de Graduados - Medellín (ANT), Colombia

**Keywords:** diabetes, habits, lifestyles, precarious word, informal jobs

## Abstract

**Introduction:**

According to American Diabetes Association, diabetes is a metabolic change characterized by the presence de hyperglycemia caused by a deficiency and/or malfunctioning of insulin secretion.

**Objectives:**

To determine sociodemographic and labor conditions, habits and lifestyles that explain diabetes in a group of informal street workers in downtown Medellín, Colombia.

**Methods:**

This is a cross-sectional study with analytical intent based on primary sources information and on a survey with a sample of 686 workers in 2016, after obtaining informed consent. Study variables included sociodemographic and labor conditions, habits, lifestyles, and diagnosis of diabetes. Univariate, bivariate and multivariate analyses were performed.

**Results:**

Workers with 50 years, 57.6% men, with a partner (56.8%), and more than 20 years in their profession. Higher prevalence of diabetes in those aged 18 to 44 and 45 to 59 years of age, lower schooling, consumed mid-morning, mid-afternoon, and evening snacks, and of households with food insecurity. Higher prevalence of diabetes was explained by: lower age, higher education, consumption of sugars, sweets, and desserts; and lower prevalence by consumption of mid-morning snacks, and household food insecurity.

**Conclusions:**

This disease of public health concern is explained by modifiable factors that can be controlled and avoided to improve the living and health conditions of this workers’ population.

## INTRODUCTION

Informal employment is one of the major sources of income for the working population of Latin America and the Caribbean (LAC),^1^ a region comprising considered developing and transition countries, and accounts for 35 to 90% of the overall employed population.^2^

Although workers’ health status has been deteriorating, this situation may be more serious for those who have subsistence jobs, i.e., who exercise their profession on streets and sidewalks,^3^ which occurs both in Colombia and in other countries.^4^ In these settings, currently there is scarce evidence showing prioritization of actions aiming to promote health and preventing diseases, particularly chronic and degenerative ones, which is reflected on the prevalence of diseases that have a negative impact on quality of life^5,6^ and on the health of workers with subsistence jobs.

Some highly-prevalent diseases among informal workers include diarrhea, pneumonia, and chronic diseases, as shown in a study with workers from a market square in Medellín.^7^ This is accompanied by a deficient health care coverage, since less than 50.0% of informal workers in Colombia integrated into the health system^8,9^ as contributing members. With regard to the specific topic of chronic and degenerative diseases, diabetes is the second chronic disease more associated with mortality from cardiovascular disease,^10^ which is why it is important to provide evidence on the risk factors that favor its onset, as well as on its prevalence, and on how these factors are associated and explain diabetes in informal workers with subsistence jobs, particularly in downtown Medellín.

Although some studies provide information for informal workers, they do not necessarily focus on those who work on streets and sidewalks, there have been reports of unhealthy life habits, such as sedentarism,^7,8,11,12^ diets with high fat and carbohydrate content,^7,8,11^ alcoholism,^7,8^ as well as comorbidities such as arterial hypertension, dyslipidemia,^7,12^ and obesity^7,11^ in the general worker population. Since it is a considerably heterogeneous population about which there is little information, it is not possible to reliably extrapolate these data to informal workers who perform their job on streets and sidewalks, especially considering that diabetes mellitus is a disease of public health interest, both because of its distribution and of its resulting chronic complications. According to American Diabetes Association, diabetes is a metabolic change characterized by the presence de hyperglycemia caused by a deficiency and/or malfunctioning of insulin secretion.^13^ Furthermore, according to the World Health Organization, in 2014 the worldwide prevalence of diabetes in individuals older than 18 years of age was 8.5%, and in 2015 there were 1.6 million deaths from diabetes.^8,14^ These are reasons that show the need to investigate which sociodemographic and labor conditions, habits, lifestyles, and comorbidities are associated with and explain the presence of diabetes mellitus in a group of informal street workers in downtown Medellín, in order to obtain information that allows to advance on the planning and implementation of actions that facilitate the proper management and prevention of a disease of public health concern in Colombia and in LAC.

## METHODS

### DESIGN

Exploratory, cross-sectional study with primary information sources, derived from the macro project of the doctoral thesis entitled “Condiciones ambientales, laborales, sociales, demográficas, económicas y de salud que determinan el perfil de vulnerabilidad laboral de un grupo de trabajadores informales ‘venteros’ del centro de Medellín 2015-2019”.

### POPULATION

Census with 686 workers, contacted by their “representative” leaders and by the main researcher at their market stalls or at meetings and guild assemblies. An assisted survey was administered at one of their guild headquarters, after informed consent was obtained from each worker. The study and all its activities were arranged with workers’ leaders and with workers themselves. Individuals older than 18 years of age and who had been working in the city downtown for at least five years were included, after being clarified about the study, its procedures, benefits, scopes, limitations, and decision to participate. No patient was excluded according to the established criteria. The study was approved by the Institutional Human Ethics Committee - Universidad CES, record No.84-code 470 of 2015.

### VARIABLES

Diabetes mellitus was considered the dependent variable, as reported by medical diagnosis. Data collection followed a previous standardization by the main researcher and a public health professional, who assisted in data collection. The independent variables were the following: sociodemographic conditions (age, sex, marital status, educational level, socioeconomic level); labor conditions (type of vendor, type of product sold, length of time in the profession); habits, lifestyles, and comorbidities (physical activity, eating habits, alcohol consumption, number of meals per day, cooking methods of foods, types of food consumed, adherence to treatment, weight self-perception, underweight and obesity, and arterial hypertension). Overweight and obesity were assessed using body mass index (BMI) and re-categorized according to standards established for nutritional use: 1. BMI < 18.5: underweight; 2. BMI 18.5-24.9: normal weight; 3. BMI 24.9-29.9: overweight; 4. BMI > 30: obesity. For bivariate and multivariate analyses, BMI was re-categorized into: 1. BMI > 24.9: overweight/obesity; and 2. BMI < 24.9: underweight/normal weight.

### ANALYSIS

A descriptive bivariate analysis was performed to explore the non-causal associations between the variables considered as explicative and prevalence of diabetes mellitus. The chi-square test of statistical association was performed, and prevalence ratios (PR) and their corresponding 95% confidence interval (95%CI) were calculated to establish the strength of association between presence de diabetes and sociodemographic variables, labor variables, habits, lifestyles, and comorbidities. The student’s t-test was used to identify differences between means for age and diabetes. All quantitative variables were re-categorized for subsequent analyses. Multivariate analyses with multiple logistic regression with explanatory purposes were performed, including explanatory variables with p < 0.25 (according to the Hosmer-lemeshow test). All tests were performed with a confidence level of 95% and a significance level of 5.0%.

## RESULTS

### SOCIODEMOGRAPHIC CHARACTERISTICS

Workers’ mean age was 50 years (±11.76), they were predominantly men (57.6%: 227), 56.8% had a partner, and mean years of schooling was five years (±3.14). Half of participants reported a monthly income of $650.000 (interquartile range: $350.000) or less, and 72.8% (499) belonged to low-low and low socioeconomic statuses (data not shown).

### LABOR CONDITIONS

Nearly 78.0% of participants were semi-stationary vendors (i.e., they occupied the public space during their working day). With regard to the type of marketed products, more than a half sold goods and pots (58.8%), followed by seasonal and perishable products (15.6%); beverages, appetizers, and desserts (10.5%), or other products (10.2%). Nearly 66.0% had been working as a vendor between 11 and 30 years, and 23.0% had been working as a vendor for more than 30 years (Table 1).

**Table 1:** Labor conditions, habits, lifestyles, and comorbidities among informal street workers participating in the study, Medellín, 2015-2019 (n = 686).

Variable	n	%
Labor conditions		
Type of vendor		
Itinerant	32	4.7
Semi-stationary	532	77.5
Stationary	122	17.8
Type of product		
Goods and pots	403	58.8
Seasonal and perishable products	107	15.6
Beverages, appetizers, and desserts	72	10.5
Fast food	33	4.8
Other	70	10.2
Length of time working in the profession		
5 to 10 years	81	11.8
11 to 20 years	257	37.5
21 to 30 years	190	27.7
> 30 years	158	23
Habits and lifestyles		
Physical activity		
Sedentary	198	28.9
Little active	191	27.8
Active	195	28.4
Very active	101	14.7
Performs sports activities		
Yes	260	37.9
No	425	62.1
Number of meals per day		
One and two	302	44.1
Three	335	48.8
More than three	48	7.0
Meals consumed daily		
Drinks	10	1.5
Breakfast	553	80.7
Mid-morning snack	24	3.5
Lunch	604	88.2
Mid-afternoon snack	42	6.1
Dinner	536	78.2
Evening snack	14	2.0
Cooking method		
Steamed	216	31.5
Roasted	280	40.8
Baked	171	24.9
Boiled	515	75.1
Fried	222	32.4
All the above	63	9.2
Stopped consuming some type of food in the last 6 months		
Yes	320	46.9
No	363	53.1
Reasons for stopping consuming a certain food		
Lack of resources	81	25.3
Health purposes	177	55.3
Change of habits	52	16.2
Activities performed while eating		
Adjustment of point of sale	52	7.6
Serving customers	457	66.8
Handling money	432	63.2
Talking with coworkers	199	29.1
Alcohol consumption		
Yes	175	25.6
No	509	74.4
Frequency of alcohol consumption		
Once a week	74	42.3
Two or three times a week	20	11.4
> 3 times a week	3	1.7
Every day	10	5.7
Food insecurity		
Food security and mild food insecurity	314	45.7
Moderate	189	27.6
Severe	183	26.8
Weight self-perception		
Underweight (thin)	65	9.5
Adequate	334	49.4
Overweight	272	40.2
Obese	6	0.9
Body mass index		
Overweight, obese	470	68.7
Underweight, obese	214	31.3
Consultation with physician		
Yes	248	86
No	40	14
Arterial hypertension		
Yes	139	48.1
No	150	51.9
Adherence to treatment		
Excellent	180	72.6
Very good	15	6.0
Good	12	4.8
Fair	22	8.9
Poor	13	5.3

### HABITS AND LIFESTYLES

Nearly half of participants had three meals (48.8%) or less a day. They predominantly had breakfast (80.7%), lunch (88.2%), and dinner (78.2%), followed by mid-morning, mid-afternoon, and evening snacks. Moreover, they preferred boiled (75.1%), roasted (40.8%), fried (32.4%), steamed (31.5%) y baked (24.9%) foods. It was observed that 27.6% (189) and 26.4% (181) of workers had moderate and severe household food insecurity, respectively, with women showing the highest rates (Table 1).

Conversely, 46.9% of participants reported to having stopped consuming some type of food in the last 6 months, mainly due to health reasons (55.3%), lack of resources (25.3%), or changes in habits (16.2%). Of the participants, 66.8% (457) reported to serving customers while eating, 63.2% (432) handled money and served customers, and 29.1% (199) talked with their coworkers while eating. Furthermore, 25.6% (175) reported to consume alcoholic beverages, of which 42.3% (74) with a frequency of once a week, 11.4% (20) of two to three times a week, and almost 8.0% (13) > 3 times a week (Table 1).

Of the participants, 49.4% (339) perceived themselves as having appropriate weight; however, 28.0% (180) were obese, and 39.5% (290) were overweight, according to BMI. Additionally, 62.1% did not perform sports activities. A total of 40.1% of participants were diagnosed with a chronic degenerative disease; of those, 48.1% presented with arterial hypertension, and 72.6% (180) reported excellent adherence to treatment for their disease (Table 1).

### SOCIODEMOGRAPHIC AND LABOR CONDITIONS ASSOCIATED WITH DIABETES

The prevalence of diabetes was almost 90.0% lower among men and among those without a partner. Those belonging to the lower socioeconomic statuses had a 30.0% lower prevalence of diabetes than those who belonged to the low-medium and medium statuses (Table 2).

**Table 2:** Sociodemographic and labor conditions associated with diabetes mellitus in informal street workers participating in the study, Medellín, 2016 (n = 686).

Characteristic	Presence de diabetes mellitus		Total	Chi-square (p-value)	PR (95%CI)
	Yes (n/%)	No (n/%)			
Sociodemographic conditions					
Marital status - Pressure					
Without a partner	43(14.5)	253(85.5)	296	0.45 (0.501)	0.88 (0.62-1.26)
With a partner	64(16.4)	326(83.6)	390		1.0
Biological sex					
Male	28 (7.1)	367 (82.9)	395	0.004 (0.94)	0.98 (0.57-1.70)
Female	21 (7.2)	270 (92.8)	291		1.0
Marital status					
Without a partner	20 (6.8)	276 (93.2)	296	0.117 (0.73)	0.91 (0.52-1.57)
With a partner	29 (7.4)	361 (92.6)	390		1.0
Age re-categorized into three groups					
60 years or over	25(16.3)	128 (83.7)	153	21.335(0.00)	1.00
18 to 44 years	3(1.6)	187 (98.4)	190		12.17 (3.6-41.17)
45 to 59 years	21 (6.7)	293 (93.3)	314		2.72 (1.47-5.05)
Socioeconomic status					
Low-low and low	32 (6.4)	468 (93.6)	500	1.534 (0.21)	0.70 (0.39-1.23)
Low-medium and medium	17 (9.1)	169(90.9)	186		1.0
Educational level					
0-5 of schooling	42 (9.6)	396 (90.4)	438	10.93 (0.001)	3.40 (1.55-7.45)
> 5 years of schooling	7 (2.8)	241 (97.2)	186		1.0
Labor conditions					
Type of vendor					
Stationary	13 (10.7)	109 (89.3)	122	2.76 (0.09)	1.67 (0.91-3.05)
Semi-stationary and itinerant	36 (6.4)	528 (93.6)	564		1.0
Type of product sold					
Dry goods/pots	28 (6.9)	375 (93.1)	403	0.056 (0.81)	0.94 (0.54-1.61)
Other types	21 (7.4)	262 (92.6)	283		1.0
Length of time in the profession divided into 2 categories					
> 20 years	29 (8.3)	319 (91.7)	348	1.509 (0.21)	1.41(0.81-2.44)
≤ 20 years	20 (5.9)	318 (94.1)	338		1.0

95%CI: 95% confidence interval; PR: prevalence ratio. Content in bold: statistically significant association when p < 0.05.

A statistically significant association (p < 0.05) was observed between age and presence of diabetes, in which for every worker with diabetes aged 60 years or older, there were 12.17 workers with diabetes from 18 to 44 years old (PR: 12.17; 95%CI: 3.60;41.17) and 2.72 workers with diabetes between 45 and 59 years old (PR: 2.72. 95%CI: 1.47;5.05). There was also a significant association (p < 0.05) between diabetes and educational level, since less educated participants presented with higher prevalence rates for the disease (PR: 3.40. 95%CI: 1.55;7.45) (Table 2).

Those who had been working as vendors for more than 20 years and who were stationary vendors had a higher prevalence of diabetes than those who did not have these characteristics, and this disease was 94.0% less prevalent in workers who sold goods and pots (Table 2).

### HABITS AND LIFESTYLES ASSOCIATED WITH DIABETES

Those who preferred roasted, baked, fried, and steamed foods had from 50.0 to 65.0% lower prevalence of diabetes than those who did not prefer these cooking methods, with a statistically significant association (p < 0.05) with regard to those who consumed fried foods (PR: 0.53. 95%CI: 0.271;057). Diabetes was also significantly more prevalent in those who had mid-morning, mid-afternoon, and evening snacks (p < 0.05), meaning that, for every worker with diabetes who did not have these meals, there were 3.13 workers with diabetes who had mid-morning snacks (PR: 3.13. 95%CI: 1.36;7.18), 2.55 who had mid-afternoon snacks (PR: 2.55. 95%CI: 1.22;5.33), and 3.12 who had evening snacks (PR: 3.12. 95%CI: 1.10;8.85). The prevalence of diabetes was also higher in those who had lunch (PR: 2.06). Conversely, those who consumed sugars, sweets, and desserts had an 89.0% lower (p < 0.05) prevalence of diabetes (PR: 0.19. 95%CI: 0.10;0.35) than those who did not consumed these foods. There was a significant lower (p < 0.05) prevalence of diabetes in those who liquor, reaching 67.0% (PR: 0.33. 95%CI: 0.13;0.82) (Table 3).

**Table 3:** Habits, lifestyles, and comorbidities associated with diabetes mellitus in informal street workers participating in the study, Medellín, 2016 (n = 686).

Characteristic	Presence of diabetes mellitus		Total	Chi-square test (p-value)	PR (95%CI)
	Yes (n/%)	No (n/%)			
Habits and lifestyles					
Cooking method					
Roasted					
Yes	15 (5.4)	265 (94.6)	280	2.33 (0.12)	0.64 (0.35-1.15)
No	34 (8.4)	370 (91.6)	404		1.0
Baked					
Yes	8 (4.7)	163 (95.3)	171	2.12 (0.14)	0.58 (0.28-1.22)
No	41 (8.0)	472 (92.0)	513		1.0
Fried					
Yes	10 (4.5)	212 (95.5)	222	3.49 (0.006)	0.53 (0.27-1.05)
No	115(24.9)	347 (75.1)	462		1.0
Steamed					
Yes	11 (5.1)	205 (94.9)	216	2.04 (0.15)	0.63 (0.33-1.20)
No	38 (8.1)	430 (91.9)	468		1.0
Meals consumed daily					
Breakfast					
Yes	40 (7.2)	513 (92.8)	553	0.03 (0.86)	1.06 (0.52-2.13)
No	9 (6.8)	123 (93.2)	132		1.0
Mid-morning snack					
Yes	5 (20.8)	19 (79.2)	24	7.01 (0.008)	3.13 (1.36-7.18)
No	44 (6.7)	617 (93.3)	661		1.0
Lunch					
Yes	46 (7.6)	558 (92.4)	604	1.65 (0.20)	2.06 (0.65-6.45)
No	3 (3.7)	78 (96.3)	81		1.0
Mid-afternoon snack					
Yes	7 (16.7)	35 (83.3)	42	6.10 (0.01)	2.55 (1.22-5.33)
No	42 (6.5)	601 (93.5)	643		1.0
Dinner					
Yes	39 (7.3)	497 (92.7)	536	0.06 (0.81)	1.08 (0.55-2.12)
No	10 (6.7)	139 (93.3)	149		1.0
Evening snack					
Yes	3 (21.4)	11 (78.6)	14	4.38 (0.03)	3.12 (1.10-8.85)
No	46 (6.9)	625 (93.1)	671		1.0
Type of food					
Tubers and bananas					
Yes	42 (7.0)	554 (93.0)	596	0.06 (0.80)	0.90 (0.42-1.95)
No	7 (7.8)	83 (92.2)	90		1.0
Fats and oils					
Yes	37 (6.5)	533 (93.5)	570	2.16 (0.14)	0.62 (0.33-1.16)
No	12 (10.3)	104 (89.7)	116		1.0
Sugars, sweets, and desserts					
Yes	13 (2.9)	435 (97.1)	448	35.02 (0.00)	0.19 (0.10-0.35)
No	36 (15.1)	202 (84.9)	238		1.0
Activities performed while eating					
Reserves exclusive time for eating					
Yes	20.98 (8.9)	204 (91.1)	224	1.56 (0.26)	1.42 (0.82-2.45)
No	29 (6.3)	431 (93.7)	460		1.0
Eats while adjusting the selling stall					
Yes	3 (5.8)	49 (94.2)	52	0.17 (0.79)	0.80 (0.45-1.36)
No	46 (7.3)	586 (92.7)	632		1.0
Eats while serving customers					
Yes	30 (6.6)	427 (93.4)	457	0.74 (0.43)	0.78 (0.45-1.36)
No	19 (8.4)	208 (91.6)	227		1.0
Eats while handling notes or coins					
Yes	26 (6.0)	406 (94.0)	432	2.31 (0.16)	0.66 (0.39-1.13)
No	23 (9.1)	229 (90.9)	252		1.0
Eats while talking with coworkers or friends					
Yes	11 (5.5)	188 (94.5)	199	1.13 (0.33)	0.71 (0.37-1.35)
No	38 (7.8)	447 (92.2)	485		
Receives treatment for his/her disease					
Yes	3 (6.1)	37 (15.5)	40	2,98 (0,084)	1,14 (1,02-1,26)
No	46 (93,9)	202 (81.5)	248		1.0
Household food insecurity					
Moderate- severe	34 (9.2)	336(90.8)	370	5.07 (0.02)	1.94 (1.07-3.49)
Mild - safe	15 (4.7)	301(95.3)	316		1.0
Consumption of liquor					
Yes	5 (2.9)	170 (97.1)	175	6.56 (0.01)	0.33 (0.13-0.82)
No	44 (8.6)	465 (91.4)	509		1.0
Physical activity					
Very active-active	15 (5.1)	282 (94.9)	297	4.56 (0.063)	1.0
Sedentary-Little active	34 (8.7)	355 (91.3)	389		1.73 (0.96-3.12)
Treatment adherence					
E, VG, G**	41 (19.8)	166 (80.2)	207	2.62 (0.270)	1.0
Fair	1 (4.5)	21 (95.5)	22		5.19 (0.68-39.70)
Poor and very poor	4 (22.2)	14 (77.8)	18		0.86 (0.27-2.76)
Weight self-perception					
Overweight - obesity	20 (7.4)	252 (92.6)	272	0.08 (0.777)	1.05 (0.74-1.49)
Underweight - appropriate weight	27 (6.8)	371 (93.2)	398		1.0
Body mass index - BMI					
Overweight-obesity	36 (7.7)	434 (92.3)	470	0.55 (0.525)	1.26 (0.68-2.33)
Overweight - Appropriate weight	13 (6.1)	201 (93.9)	214		1.0
Presence of arterial hypertension					
Yes	30 (61.2)	109 (17.1)	139	54.80 (0.000)	0.16 (0.09-0.28)
No	19 (38.8)	528 (82.95)	547		1.0

E, VG,G: excellent, very good, good; 95%CI: confidence interval; PR: prevalence ratio. Content in bold: statistically significant association with p < 0.05.

There were statistically significant associations indicating higher or lower presence of diabetes among those who combined eating with other activities. However, the presence of diabetes was 34.0% lower among those who reserved exclusive time for eating compared with those who handled notes or coins while eating. Conversely, household food insecurity was significantly associated (p < 0.00) with prevalence of diabetes, meaning that those experience moderate or severe food insecurity had a 94.0% higher prevalence of the disease (PR: 1.94. 95%CI: 1.07;3.49) (Table 3).

It was found that the higher self-perception of overweight and obesity, the lower the prevalence of diabetes. However, when relating the prevalence of diabetes with BMI, it was found that, for each underweight or normal weight worker with diabetes, there was 1.26 overweight or obese worker with this disease. Those reporting to be sedentary or little active had a 73.0% higher prevalence of diabetes than those who reported to be active or very active. There was also a statistically significant association (p < 0.05) between arterial hypertension and diabetes, with hypertensive individuals having a 84.0% lower prevalence of diabetes than those with no arterial hypertension (PR: 0.16, 95%CI: 0.09;0.28). It was also observed that, for each worker with diabetes who considered having a very food or good adherence to treatment for diabetes, there were 5.19 workers with diabetes who had fair treatment adherence (Table 3).

### CONDITIONS THAT EXPLAIN THE PREVALENCE OF DIABETES AMONG WORKERS

One of the factors that were able to significantly explain the disease (p < 0.05) in informal street workers in downtown Medellín were age, which maintained its statistical significance and direction of association in multivariate analysis, meaning that, for each worker with diabetes aged 60 years or older, there were 5.43 workers with diabetes aged from 18 to 44 years old (PR_unadjusted_: 12.17, 95%CI: 3.60;41.17. PR_adjusted_: 5.43, 95%CI: 1.25;23.63), and 2.17 aged from 45 y 59 years old. The latter age group, despite losing its statistical significance, maintained its direction of association (Table 4).

**Table 4:** Sociodemographic and labor conditions, habits, lifestyles, and comorbidities that explain the prevalence of diabetes in informal street workers participating in the study, Medellín, 2016. (n = 686).

Condition - characteristic	Unadjusted PR	95%CI		Adjusted PR	95%CI	
		LT	UT		LT	UT
Sociodemographic conditions						
Re-categorized age. 60 or older (Rc)						
18 to 44 years	12.17	3.60	41.17	5.43	1.25	23.63
45 to 59 years	2.72	1.47	5.05	2.17	0.99	4.78
Socioeconomic status. Low-medium and medium (Rc)						
Low-low and low	0.70	0.39	1.23	1.55	0.71	3.41
Educational level. More than 5 years of schooling (Rc)						
0-5 years of schooling	3.40	1.55	7.45	0.32	0.11	0.90
Labor conditions						
Type of vendor. Semi-stationary and itinerant (Rc)						
Stationary	1.67	0.91	3.05	1.56	0.66	3.70
Length of time in the profession. ≤ 20 years (Rc)						
> 20 years	1.41	0.81	2.44	1.49	0.66	3.38
Habits and lifestyle - cooking methods						
Roasted. No (Rc)						
Yes	0.64	0.35	1.15	1.88	0.47	7.55
Baked. No (Rc)						
Yes	0.58	0.28	1.22	1.44	0.50	4.13
Fried. No (Rc)						
Yes	0.53	0.27	1.05	0.66	0.13	3.45
Steamed. No (Rc)						
Yes	0.63	0.33	1.20	0.65	0.24	1.76
Meals consumed daily						
Mid-morning snack. No (Rc)						
Yes	3.13	1.36	7.18	0.13	0.02	0.76
Lunch. No (Rc)						
Yes	2.06	0.65	6.45	0.55	0.15	2.06
Mid-afternoon snack. No (Rc)						
Yes	2.55	1.22	5.33	1.68	0.39	7.23
Evening snack. No (Rc)						
Yes	3.12	1.10	8.85	0.37	0.05	2.85
Type of food						
Fats and oils. No (Rc)						
Yes	0.62	0.33	1.16	1.03	0.43	2.49
Sugars, sweets, and desserts. No (Rc)						
Yes	0.19	0.10	0.35	5.48	2.44	12.31
Activities performed while eating						
Eats while handling notes or coins. No (Rc)						
Si	0.66	0.39	1.13	1.71	0.80	3.66
Receives treatment. No (Rc)						
Si	1.14	1.02	1.26	0.54	0.13	2.23
Household food insecurity. Mild - food security (Rc)						
Moderate - severe food insecurity	1.94	1.07	3.49	0.43	0.19	0.98
Consumption of liquor. No (Rc)						
Yes	0.33	0.13	0.82	1.09	0.37	3.22
Physical activity. Very active - active (Rc)						
Sedentary - Little active	1.73	0.96	3.12	0.61	0.28	1.35
Presence of arterial hypertension. No (Rc)						
Yes	0.16	0.09	0.28	0.82	0.38	1.76

95%CI: confidence interval; LT: lower threshold; UT: upper threshold; PR: prevalence ratio. Rc: reference category for comparison. The results are presented for the categories to which this category is compared. Content in bold: statistically significant association when p < 0.05.

The greater prevalence of diabetes was also explained by the consumption of sugars, sweets, and desserts, since those who consumed this type of food had a 4.48-fold higher prevalence of diabetes compared to those who did not consume them. It is important to bear in mind that this habit was found to be a protective factor, but recovered its explanatory capacity and changed its direction of association when adjusted for the remaining variables in the analysis, as a risk factor (PR_unadjusted_: 0.19, 95%CI: 0.10;0.35. PR_adjusted_: 5.48, 95%CI: 2.44;12.31) (Table 4).

Conversely, the following factors were able to significantly explain a lower prevalence of diabetes; having from zero to five years of schooling, having mid-morning snacks, and living in households with moderate or severe food insecurity. When adjusting these conditions for the remaining variables, they changed their direction of association and their explanatory capacity, changing from being associated to a higher prevalence of diabetes to explaining a lower prevalence of this disease, since the prevalence of diabetes was 68.0% lower among those with lower educational level (PR_adjusted_: 3.40, 95%CI: 1.55;7.45. PR_adjusted_: 0.32, 95%CI: 0.11;0.90), and 87.0% lower among those who did not have mid-morning snacks every day (PR_unadjusted_: 3.13, 95%CI: 1.36;7.18. PR_adjusted_: 0.13, 95%CI: 0.02;0.76). Having lunch and evening snack daily were not able to significantly explain the disease; however, when adjusted for the remaining variables changing from being associated with a higher prevalence of diabetes to explaining its lower prevalence (lunch PR_adjusted_: 0.55; evening snack PR_adjusted_: 0.37). Having mid-afternoon and evening snacks every day lost its significance; however, those who had mid-afternoon snacks had a 68.0% higher prevalence of diabetes (PR_adjusted_: 1.68) than those who did not. Conversely, those who reported moderate or severity household food insecurity had a 57.0% lower prevalence of diabetes than those who reported food security or mild food insecurity (PR_unadjusted_: 1.94, 95%CI: 1.07;3.49. PR_adjusted_: 0.43, 95%CI: 0.19;0.98) (Table 4).

Living in low-low or low socioeconomic status households changes from being associated with lower presence of diabetes to explaining a higher prevalence of this disease (PR_adjusted_: 1.55). Similar situation was found regarding the consumption of roasted and baked foods, which allowed to explain 88.0% and 44.0% of higher prevalence of the disease (PR_adjusted_: 1.88 and PR_adjusted_: 1.44), however, the fact of consuming fried (PR_adjusted_: 0.66) and steamed (PR_adjusted_: 0.65) foods maintained their direction of association, being able to explain a lower prevalence of diabetes (Table 4).

Labor conditions that remained associated with a higher prevalence of diabetes were: being a stationary vendor (PR_adjusted_: 1.56) and working as a vendor for more than 20 years (PR_adjusted_: 1.49), however, the fact of handling notes or coins while eating changed from being associated with lower presence of diabetes to being able to explain its higher prevalence (PR_unadjusted_: 0.66; PR_adjusted_: 1.71) (Table 4).

## DISCUSSION

Workers’ age observed in this study was similar to that reported in a Spanish study that evaluated the health-related quality of life in women working in the fishing industry, whose mean age was 50.6 years (±8.8),^15^ but was different from that reported in other studies assessing informal workers in Colombia, such as street vendors, with mean age of 26 years,^16^ and coffee collectors, who were mainly aged from 20 and 29 years old.^17^ Workers from 18 to 44 years of age showed a higher prevalence of diabetes, followed by those from 45 and 59 years of age, which revealed a statistically significant association consistent with findings reported by Ahsner et al. in 1995, who stated that the population with diabetes was increasingly younger.^18^

Workers participating in the present sample were predominantly man, a situation similar to that of a study on informal workers’ working conditions and health status in a market in Cartagena, Colombia.^19^ However, the highest percentage of diabetes in this study was observed among women, in line with the study Carmela, which was conducted in seven Latin American cities in 2005.^20^

Those reporting to have a partner had a higher prevalence of diabetes, contrary to the results for informal coffee collectors in a municipality in Colombia, which revealed that most workers were single.^17^ These workers had only five years of schooling (±3.14), different from those reported for workers from the market in Cartagena, Colombia,^19^ of which 36.4% had completed high school.^19^ Educational level was significantly associated with the prevalence of diabetes, meaning that the lower the educational level, the higher the prevalence. However, when adjusted for the remaining variables, a higher prevalence of diabetes was explained by higher educational level, differently from what was observed in a study with civil servants in Brazil, which found that less educated individuals were more prone to diabetes.^21^

Half of the workers reported monthly incomes of $650.000 or less, and 72.8% lived in low-low and low socioeconomic strata households; similar to results for vendors in a study conducted in Cartagena, Colombia,^19^ who earned $22.577 a day and lived mainly in low strata households. These findings were also similar to those from a study conducted in a hospital in Bogotá, Colombia,^22^ in which 71.3% of patients hospitalized with diabetes belonged to low strata. It is worth noting that, in the present study, belonging to the low-medium and medium socioeconomic strata to explain a higher presence of diabetes.

With regard to food insecurity in workers’ households, the results of the present study coincide with those of a study conducted in Medellín, which showed that individuals in the lower socioeconomic strata had a higher prevalence of food insecurity.^23^ Moreover, half of the respondents reported moderate or severe household food insecurity; noticeably, they showed a significantly lower prevalence of diabetes. Although few studies with informal workers have assessed the type of activities informal workers perform while eating, the present study revealed a higher presence of diabetes among those who handled notes or coins while eating.

Most participants worked for more than 11 years as vendors, corroborating data reported by the Colombian National Survey on Health and Work Conditions in the Informal Economy.^9^ It was found that they were mostly semi-stationary vendors, sold mainly goods and pots, and that, the longer the length of time in the profession, the higher the prevalence of diabetes. In the present study, this disease was associated with or explained not only by lower age, but also by length of time in the profession, as well as by other labor conditions, habits, and lifestyles.

With regard to the meals consumed daily, the most consumed ones were breakfast, lunch, and dinner, with lunch having the highest prevalence of consumption, which in turn was associated a higher prevalence of diabetes, a situation different to that reported in a study conducted in Chile^11^ that evaluated construction workers and showed that the least consumed meal was dinner.

The foods most consumed by these workers were fats and oils, contrary to the findings reported in the Chilean study, which found that the most consumed food included fruits and vegetables.^11^ The consumed of fats and oils explained a higher prevalence of diabetes, as well the consumption of sugars, sweets, and desserts, being able to significantly explain the disease, similar to the results of a study that assessed an European adult population, which revealed that consuming 336 grams of sugar per day was associated with a higher presence of diabetes.^24^ Conversely, the prevalence of diabetes was lower for those who consumed tubers and bananas, consistent with what was found in an Iran study reporting that the daily consumption of potatoes was associated with lower risk of diabetes.^25^

The cooking methods more frequently reported by workers were boiled, roasted, fried, streamed, and baked, with the prevalence of diabetes being higher for those who consumed roasted and baked foods and lower for those who preferred fried and steamed food. These findings were different from those reported in an American study, which found that those who consumed fried food more than once a week had a 39.0 to 55.0% higher prevalence of diabetes.^26^

A total of 25.6% of workers reported consuming alcohol, mostly of them once a week; this consumption was associated with a higher prevalence of diabetes, in line with a Swedish study that showed an increased risk of diabetes among men who consumed alcohol.^27^

A total of 68.7% of workers were overweight or obese, although less than a half perceived themselves as having these condition; a situation similar to that reported in the 2007 Colombian National Health Survey.^28^ This condition was associated with a higher presence of diabetes, corroborating the results of a study that assessed a population of outpatient individuals with overweight or obesity in Spain, which had a 17.3% and 34.8% greater risk of diabetes, respectively^29^ than those with normal weight. More than a half of informal street workers reported not performing any sports activity and also being sedentary or little active. These conditions have been found as risk factors for the presence of diabetes, similar to what has been shown in a study with civil servants in Brazil, which revealed that individuals with hypercholesterolemia and diabetes simultaneously were more likely to be little active.^21^ However, it is worth noting that, in the present study, little active or sedentary workers exhibited a lower prevalence of diabetes.

The percentage of workers who did not see a doctor was 14.0%, similar to that described in a Spanish study with overweight or obese participants, of which 13.9% did not attend their follow-up appointments.^29^ Although half of workers diagnosed with a chronic, degenerative disease had arterial hypertension, it was worth noting that this condition was associated with a lower prevalence of diabetes, contrary to what was observed in the Spanish study, in which increased blood pressure was el second risk factor more associated with development of diabetes.^29^

Most participants reported excellent treatment adherence, consistent with results shown in a study conducted in Madrid to assess treatment adherence in a population of public administration workers, of which 86.0% took their medications for the management of chronic diseases.^30^ It was revealed that the fact of receiving treatment was associated with lower presence of diabetes.

It is important to bear in mind that, despite the study population consisted of 686 workers, this is a census of vendors who are members of five workers’ associations in city downtown; thus, study results should be considered for this population and, although street vendors in downtown Medellín usually share most conditions investigated here, caution is needed when analyzing and interpreting results. It is also important to bear in mind that data for diagnosis of diabetes were provided by workers themselves.

## CONCLUSION

In conclusion, a higher prevalence of diabetes in this workers’ population was explained by age from 18 to 44 years old and consumption of sugars, sweets, and desserts. Conversely, a lower prevalence of this disease was explained by having less than five years of schooling, having mid-morning snacks, and experiencing moderate or severe food insecurity. Although the literature reports that the greater the age, the higher the prevalence of diabetes, the present study found that the presence of this disease was higher in workers aged from 18 to 49 years old and a lower in older adults.

Some of these characteristics condition socialoccupational vulnerability and, except for age, could be reverted with the improvement of workers’ conditions of life and work. The present study provides information that allows for advancing the planning and implementation of actions to facilitate the appropriate management and prevention of a disease of public health concern in Colombia and in LAC.
